# Pan-cancer analysis suggests that LY6H is a potential biomarker of diagnosis, immunoinfiltration, and prognosis

**DOI:** 10.7150/jca.98449

**Published:** 2024-08-26

**Authors:** Haifei Qin, Honglong Lu, Chongjiu Qin, Xinlei Huang, Kai Peng, Yuhua Li, Chenlu Lan, Aoyang Bi, Zaida Huang, Yongguang Wei, Xiwen Liao, Tao Peng, Guangzhi Zhu

**Affiliations:** 1 Department of Hepatobiliary Surgery, The First Affiliated Hospital of Guangxi Medical University, Nanning, Guangxi Zhuang Autonomous Region, People's Republic of China.; 2 Key Laboratory of High-Incidence-Tumor Prevention & Treatment (Guangxi Medical University), Ministry of Education, Nanning 530021, People's Republic of China.

**Keywords:** LY6H, pan-cancer, diagnosis, immune infiltration, prognosis

## Abstract

LY6H, a member of the lymphocyte antigen-6(LY6) gene family, is located on human chromosomes 6, 8, 11 and 19. This superfamily is characterized by the presence of LU domains. It has demonstrated its emerging significance in various cancers including adenocarcinoma, bladder cancer, ovarian cancer and skin cancer. However, comprehensive pan-cancer analyses have not been conducted to investigate its role in diagnosis, prognosis and immunological prediction. By conducting comprehensive analysis of patient data obtained from publicly available databases, including The Cancer Genome Atlas (TCGA), Genotype-Tissue Expression (GTEx), University of Alabama at Birmingham (UALCAN), The Comparative Toxicological Genomics Database (CTD), cBioportal, cancerSEA, and UCSC, we systematically investigated the differential expression of LY6H in 33 different types of human tumors. Additionally, we thoroughly analyzed the diagnostic, prognostic, and immunoinfiltration value of LY6H. Simultaneously, we examined the correlation between LY6H and tumor stemness, methylation patterns, drug sensitivity, gene alterations as well as single cell functions. Furthermore, protein-protein interaction networks and gene-gene interaction networks for LY6H were constructed. Moreover, we also explored the network relationship between LY6H and chemical compounds or genes. The results revealed that LY6H exhibited high expression levels in most cancers which were further validated through Reverse Transcription-Polymerase Chain Reaction (RT-PCR) and Immunohistochemistry (IHC) analysis using Hepatocellular carcinoma (HCC) samples. Moreover, LY6H displayed early diagnostic potential in 12 tumors while also showing positive or negative correlations with prognosis across different tumor types. Additionally, it was found that LY6H played a pivotal role in regulating immune-infiltrating cells across multiple cancers whereas the correlation between LY6H expression and immune-related genes varied depending on their specific types. Furthermore, the expression of LY6H was significantly associated with DNA methylation patterns in 21 cancers. Therefore, LY6H could serve as an adjunctive biomarker for early tumor detection as well as a prognostic indicator for diverse malignancies.

## Introduction

The global burden of cancer on both the economy and society is substantial. Based on GLOBOCAN estimates, which analyze the incidence and mortality rates of 36 types of cancer across 185 countries, there were approximately 19.3 million new cases and nearly 10 million deaths worldwide in 2020 alone^1^. Despite the availability of various cancer treatments, including radiotherapy, chemotherapy, surgery, targeted therapy, and immunotherapy, their efficacy and prognosis remain unsatisfactory. Cancer continues to be the leading cause of human mortality and poses a significant impediment to global efforts in extending life expectancy^2^. The purpose of pan-cancer analysis is to identify commonly differentially expressed genes across various types of cancers using public databases such as TCGA and GTEx. This analysis aims to discover valuable indicators for clinical diagnosis, prognosis, and treatment. By leveraging pan-cancer analysis, we can investigate the immune infiltration patterns of specific genes in diverse cancers, thereby providing crucial insights for cancer therapy^3^, ^4^.

LY6H is a member of the lymphocyte antigen-6 (LY6) gene family, which is located on human chromosomes 6, 8, 11 and 19. This superfamily is characterized by the presence of LU domains^5^. The LU domains consist of an 80-amino acid domain containing ten cysteines arranged in a specific spacing pattern that allows for different disulfide bonds, resulting in a three-finger (3F) structural motif^6^. The LU domains exhibit topological similarity to the three-finger structure found in snake venom neurotoxins and are held together by their unique disulfide bonds, forming three β-sheet loops^7^. Ly6H protein displays diverse expression patterns and plays crucial roles in cell proliferation, migration, cell-cell interactions, immune cell maturation, macrophage activation, and cytokine production. These effects are typically mediated through its interaction with nicotinic acetylcholine receptors^8^.

Previous studies have shown that the LY6 family and c-Myc are located together on chromosome 8q24 and that somatic copy number gain at 8q is associated with the most prevalent copy number gain in multiple cancer types^9^. The literature convincingly demonstrates that elevated levels of LY6 family genes disrupt TGF-β signaling and inhibit GDF10 expression, thereby promoting breast tumorigenesis in mouse models^10^. In recent years, the LY6H gene has garnered increasing attention due to its multifaceted involvement in cancer development, stem cell maintenance, immune regulation, and association with more aggressive and refractory cancers. The LYPD3 domain of this gene, a glycosylphosphatidylinositol-anchored molecule, has been found to be highly expressed in various human cancers including urothelial carcinoma, breast cancer, lung cancer and esophageal carcinoma. Moreover, LYPD3 is expressed in over 80% of primary colorectal tumors as well as liver metastases and specifically localized at the invasive front where it is associated with epithelial-mesenchymal transition (EMT)^6^.

The present study employed a comprehensive bioinformatics analysis to elucidate the association between LY6H expression and the diagnosis, prognosis, as well as immune infiltration in various cancer types. By utilizing patient data from multiple databases, we conducted analyses on immune infiltration, single-cell functionality, methylation patterns, drug sensitivity profiles, gene enrichment pathways; furthermore, we explored genetic alterations of LY6H and its correlation with tumor stemness. Additionally, GGI and PPI interaction networks were constructed. Finally, RT-PCR was performed to validate the differential expression of LY6H in HCC samples. In conclusion, our findings suggest that LY6H holds potential as a pan-oncogene and an immune infiltration-related biomarker particularly in HCC.

## Materials and Methods

### Data collection

The mRNA expression levels and clinical data of 33 kinds of cancer tissues and corresponding adjacent tissues from TCGA database and GTEx database were downloaded and analyzed. The pair analysis of 33 kinds of cancer tissue and normal tissue was carried out. Log2 transformation method was used to identify the expression difference between 33 tumor tissues and neighboring tissues. The abbreviations for the 33 types of cancer are provided in the Abbreviations section.

### Analysis of diagnostic value of LY6H

We used the "ggplot2" R package to extract the Clinical features and tumor stages of 33 TCGA tumors, and analyzed their relationship with LY6H expression. A receiver operating characteristic ROC curve study of LY6H diagnostic values for 33 tumors was performed using the "pROC" R package, and the area under the curve (AUC) for each tumor was calculated, ranging from 1.0 (fully diagnosed) to 0.5 (undiagnosed).

### Analysis of prognostic value of LY6H

We obtained survival data corresponding to 33 cancers from the samples downloaded by TCGA. Patients were divided into high/low expression groups based on the median LY6H expression. COX regression analysis was used to investigate the correlation between LY6H and the prognosis of patients, and the results were presented in the form of forest map. Survival analysis was performed for each cancer using Kaplan-Meier method and logarithmic rank sum test, and survival curves were plotted using the "survival" and "survminer" R packages. The observed indicators are OS, DSS, and PFI.

### Relationship between LY6H and immune infiltration

We downloaded the standardized pan-cancer dataset from the UCSC (https://xenabrowser.net/) database: TCGA TARGET GTEx (PANCAN, N=19131, G=60499), we further extracted the expression data of LY6H gene in each sample, and further carried out log2(x+0.001) transformation for each expression value. In addition, we also extracted the gene expression profile of each tumor. The expression profile is mapped to GeneSymbol, further utilizing the R software package “IOBR” deconvo_xCell methods, Infiltration scores for each patient and 67 classes of immune cells in each tumor were re-evaluated based on gene expression. We used the “corr.test” function of R package “psych”(version 2.1.6) to calculate Pearson's correlation coefficient of gene and immune cell infiltration score in each tumor to determine the immunoinfiltration score that was significantly associated.

We used the R software package "ESTIMATE" to calculate stromal, immune, and estimate scores for each patient in each tumor based on gene expression. Further, we extracted the expression data of LY6H gene and 150 marker genes of five types of immune pathways (chemokine (41), receptor (18), MHC (21), Immunoinhibitor (24) and Immunostimulator (46)) in each sample. Next, we calculated the pearson correlation between LY6H and five immune pathway marker genes. In addition, expression data of LY6H gene and 60 marker genes of two types of immune checkpoint pathway genes (Inhibitory (24) and Stimulatory (36) in each sample were extracted, and pearson correlation between LY6H and immune checkpoint genes was calculated.

### Correlation of LY6H with TMB, MSI, MATH gene and tumor stemness

By exploring the correlation between LY6H and TMB gene, MSI gene and MATH gene, the relationship between LY6H and tumor mutation load, microsatellite instability and tumor heterogeneity of allelic mutation was obtained, so as to further study the role of LY6H in immunotherapy. In addition, we further investigated the correlation between LY6H and tumor stemness. The results were obtained by Pearson correlation calculation.

### LY6H expression and methylation analysis

We used the UALCAN database to study the promoter methylation level of LY6H. The UALCAN database is an open, interactive portal that integrates publicly shared databases including TCGA and CBTN for methylation analysis. In addition, we used the DNA methylation pattern survival analysis tool MethSurv to analyze the relationship between LY6H and DNA methylation.

### Construction of PPI and GGI interaction network

STRING is a free online publicly shared database with the ability to explore gene and protein interactions and map PPI interaction networks. We used the STRING platform to analyze protein interactions and obtained protein-protein interaction (PPI) networks for LY6H protein and 10 related proteins.

We used the GeneMANIA database to explore correlations between LY6H and its similar genes and construct gene-gene interaction^11^ networks. GeneMANIA is a completely free, user-friendly website that uses a wealth of genomic and proteomic data to find functionally similar genes and further analyze the relationship between genes and genes^12^, ^13^. In conclusion, LY6H is intricately associated with the occurrence and progression of a diverse range of tumors; however, further investigation is required to elucidate the underlying mechanisms.

### Drug sensitivity analysis

We used the GSCALite database to study the drug-sensitive correlation of LY6H and its related genes in different tumor species. GSCALite is a fully open gene-set cancer analysis Web server for analyzing cancer gene and drug sensitivity correlations^14^.

### Expression of LY6 in cancer molecular subtypes and immune subtypes

We used the TISIDB database to explore the correlation between LY6H expression and pancarcinoma molecular subtypes and immune subtypes. The TISIDB database is a user-open platform through which users can analyze the role of genes of interest in tumor-immune interactions^15^.

### Genetic alteration of LY6H in pancarcinoma

We explored evidence of LY6H gene mutations from the cBioPortal database with the goal of understanding LY6H changes in pancarcinoma. cBioPortal for Cancer Genomics (http://cbioportal.org) is a comprehensive online platform for the exploration, visualization and analysis of multi-dimensional cancer genomic data^16^.

### Single cell function analysis of LY6H

We performed single-cell function analysis of LY6H using the cancerSEA database. The CancerSEA database (http://biocc.hrbmu.edu.cn/CancerSEA/) is the pioneering resource that investigates the comprehensive functional states of cancer cells at a single-cell level^17^.

### LY6H interacts with chemicals and genes

We used the CDC database to study the interactions of LY6H with chemicals and used the database to explore genes that are highly similar to LY6H in common interacting chemicals. The Comparative Toxicological Genomics Database (CTD, http://ctdbase.org/) is a cutting-edge digital platform that facilitates the integration of toxicological data related to chemicals, genes, phenotypes, diseases, and exposures in order to enhance comprehension of human health^18^.

### Identification of DEGs and Functional enrichment analysis in LIHC

We employed the R package "pheatmap" to perform differential gene expression analysis and generate heat maps. We utilized the R package "ClusterProfiler (version 3.14.3)" to perform gene enrichment analysis, aiming to identify pathways and biological functions associated with DEGs (differentially expressed genes) and LY6H in LIHC. Gene ontology (GO) terms and Kyoto Encyclopedia of Genes and Genomes (KEGG) pathway were employed for gene annotation. The obtained results were visualized using the "ggplot2" package, considering adjusted p-values below 0.05 and an error Finding rate (FDR) less than 0.25 as indicators of significance.

### RT-PCR verification of LY6H expression in LIHC

To verify the differential expression of LY6H in cancer tissues and adjacent tissues, PCR was performed on postoperative specimens of 24 pairs of HCC patients in the Guangxi cohort of the First Affiliated Hospital of Guangxi Medical University. The patients included in this study were exclusively diagnosed with HCC and did not present any comorbidities. The supplementary material (table S1) includes a list of 24 patients with clinical parameters. RNA was isolated with Trizol reagent (Invitrogen, USA) and reverse transcribed into cDNA with PrimeScript™RT kit (Takara, China). Subsequently, FastStart Universal SYBR Green Master Mix (Roche, Germany) was used for PCR analysis, and the relative expression of LY6H mRNA was determined by 2^-∆∆CT^ method. The priming sequence used in this experiment was: internal reference gene GAPDH, forward: GTCAGCCGCATCTTCTTT, reverse: CGCCCAATACGACCAAAT; Target gene LY6H, positive: GCACCTGCACTCCCCG, reverse: ACAGGCCATGAGCGGGC. This study has been approved by the Ethics Review Committee of the First Affiliated Hospital of Guangxi Medical University [Approval No.: 2024-E302-01].

### IHC verification of LY6H expression in LIHC

Immunohistochemical analysis was performed with the use of the patient tissue specimens listed in the Supplementary material (Table S1). We selected normal liver tissue from patients with liver cancer and liver tumor tissue for paraffin embedding treatment to prepare sections. The slides were subjected to xylene dewaxing, anhydrous ethanol hydration, and antigen retrieval. Endogenous peroxidase blocking was performed for 10 minutes, followed by incubation with LY6H primary antibody (1:150, Proteintch, China) at 4°C overnight. On the second day, the specimen was treated with a reaction enhancement solution for 20 minutes. LY6H secondary antibody was added and incubated for 30 minutes. Subsequently, DAB reagent was applied for 5 minutes followed by restaining with hematoxylin. Finally, the sections were dehydrated and sealed with neutral resin. The results demonstrated that LY6H staining predominantly localized in the cytoplasm. Five random fields of view were selected for observation and scoring purposes. Expression intensity scores ranged from 0 to 3 indicating negative staining or weak (light yellow), moderate (light brown), and strong (dark brown) staining respectively. The expression area score ranged from 0 to 4 representing <5%, 6-25%,26-50%,51-75%, and >75% respectively.The degree of positive staining is defined as follows: weak positive (+) if scored between 1-3; moderately positive (++) if scored between4-6; strongly positive (+++) if scored between7-12^19^, ^20^. This study has been approved by the Ethics Review Committee of the First Affiliated Hospital of Guangxi Medical University [Approval No.: 2024-E302-01].

### Statistical analysis

We used R software (version 4.2.1) for data analysis and visualization tasks. Wilcoxon rank sum test was used to evaluate the correlation between LY6H expression and cancer/normal tissue and clinicopathological features. In the validation phase of liver cancer samples, we assessed the normality of PCR and IHC output results and applied logarithmic transformations to data that deviated from a normal distribution. Subsequently, student t-tests or paired t-tests were conducted. The Mann-Whitney U rank sum test was utilized to determine differences in data with skewed distributions. Chi-square test and Yates correction were used to compare categorical variables between groups. Correlation analysis was performed by pearson or spearman method. p < 0.05 was considered statistically significant (ns, p > 0.05; *, p < 0.05; **, p < 0.01; ***, p < 0.001, ****, p < 0.0001).

## Results

### Differential expression of LY6H in tumor and normal tissue samples

**Figure 1:**
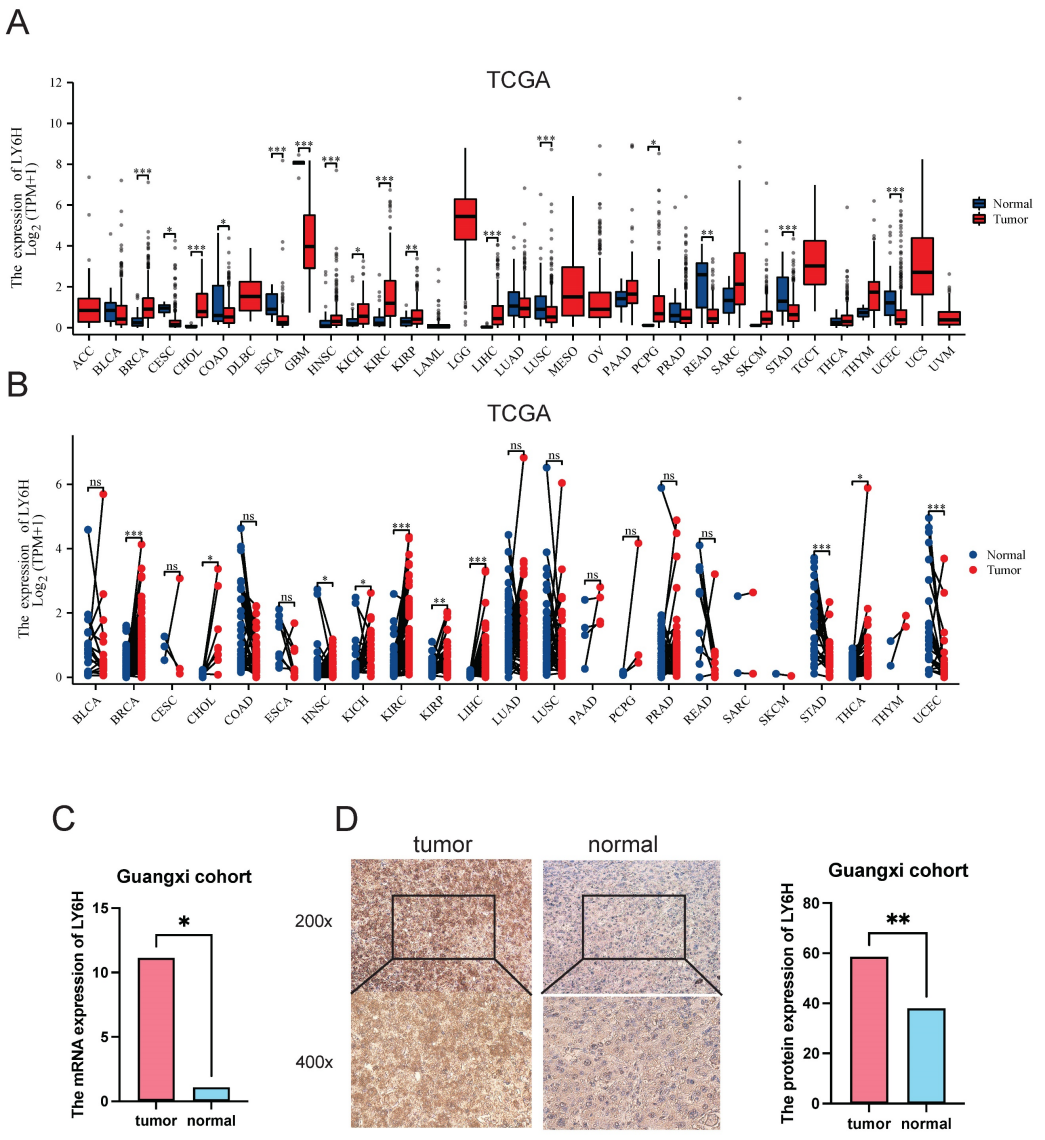
Differential Expression of LY6H. **(A)** Comparison of LY6H expression between tumor and normal samples. **(B)** comparison of LY6H expression between tumor and paired normal samples.** (C)** Differential expression of 24 pairs of HCC tissues/paracancerous tissues in the Guangxi cohort. **(D)** Typical IHC images and differential expression bar graphs. LY6H was strongly expressed in the cytoplasm of tumor tissue.

Based on the TCGA data we downloaded, we compared LY6H expression levels in 33 cancer patients with matched normal samples, excluding those with no normal tissue data or with very few normal samples (Figure 1A). We detected significant differences in LY6H expression from normal tissues in 16 cancers. Among them, LY6H has been found in bladder urothelial carcinoma (BLCA), cholangiocarcinoma (CHOL), glioblastoma multiforma (GBM), head and neck squamous cell carcinoma (HNSC), kidney chromophilous cell carcinoma (KICH), kidney clear cell carcinoma (KIRC), kidney papillary cell carcinoma^21^, primary liver cancer (LIHC), lung squamous cell carcinoma (LSSC), pheochromocytoma and paraganglioma (PCPG) are expressed higher in tumors than in normal tissues. In contrast, LY6H expression is lower in cervical squamous cell carcinoma and endometrial adenocarcinoma (CESC), colon cancer (COAD), esophageal cancer (ESCA), rectal adenocarcinoma (READ), gastric adenocarcinoma (STAD), and endometrial cancer (UCEC) than in normal tissues. In addition, compared to the corresponding normal sample, In the matched tumor samples, bladder urothelial carcinoma (BLCA), cholangiocarcinoma (CHOL), head and neck squamous cell carcinoma (HNSC), renal phobopic cell carcinoma(KICH), renal clear cell carcinoma (KIRC), renal papillary cell carcinoma^21^, primary liver cancer (LIHC), endometrial adenocarcinoma of cervix (CESC), and thyroid cancer (THCA) were selected The expression of LY6H in all 9 cancers was significantly increased; conversely, the expression of LY6H in gastric adenocarcinoma(STAD) in paired tumor samples was significantly decreased (Figure 1B). In addition, we performed RT-PCR (Figure 1C) and IHC (Figure 1D) validation in LIHC, and the results were completely consistent with our expectation. These results show that LY6H expression varies significantly across various types of cancer, suggesting that LY6H may play a potentially critical role in cancer diagnosis.

### Diagnostic value of LY6H in various cancers

**Figure 2:**
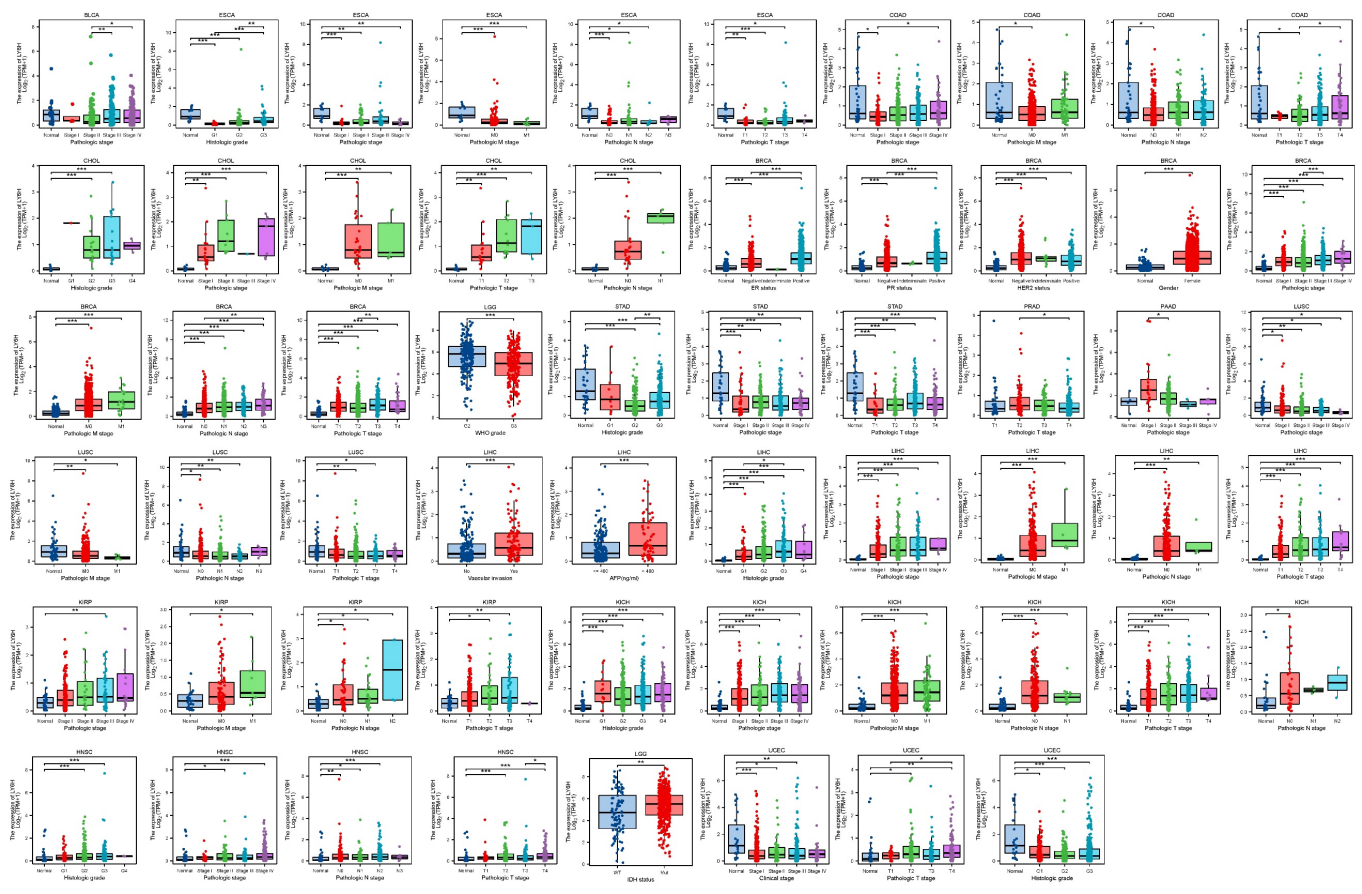
Correlation between LY6H expression and Clinical features. *p < 0.05, **p < 0.01, ***p < 0.001. ns, not statistically significant.

By conducting an analysis on the correlation between LY6H expression and clinical characteristics, we observed (Figure 2) that LY6H expression was significantly upregulated in the early stages of six tumors, namely CHOL, BRCA, LIHC, KIRP, KICH, HNSC and PAAD. Conversely, LY6H expression showed a significant decrease in the early stages of seven tumors including BLCA, ESCA, COAD, LGG, LUSC STAD and UCEC. Regarding histological grade association, LY6H expression exhibited a notable increase in the initial histological stage of four tumors: CHOL, LIHC, KICH and HNSC; conversely it displayed a significant decrease in the early histological stage of three tumors: ESCA, STAD and UCEC. In terms of BRCA specifically, the elevated expression level of LY6H was found to be associated with ER, PR, HER2 gene activity. Additionally for LIHC, the increased presence of LY6H demonstrated a strong correlation with vascular invasion as well as AFP levels. These findings suggest that LY6H may hold crucial clinical value for early tumor diagnosis.

**Figure 3:**
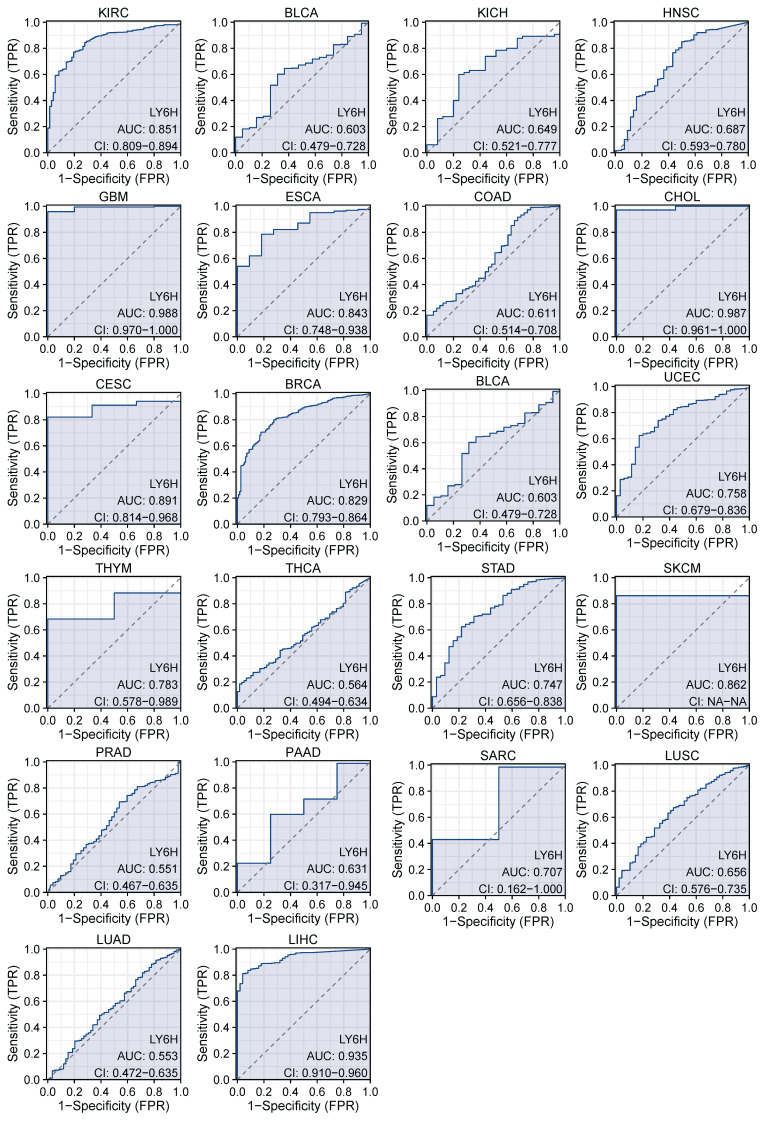
AUC of the ROC curve verified the diagnostic performance of LY6H in the TCGA cohort.

We used ROC curves to assess the diagnostic accuracy of genetic markers (Figure 3). After our study, the ROC analysis AUC of this model has a high diagnostic accuracy for 3 cancers, including GBM, CHOL and LIHC. The diagnostic accuracy was relatively high for 9 types of cancer, including KIRC, ESCS, CESC, BRCA, UCEC, THYM, STAD, SKCM, SARC. Diagnostic accuracy was low for 10 types of cancer, including BLCA, KICL, HNSC, COAD, BLCA, THCA, PRAD, PAAD, LUSC, LUAD. AUC: 1.0-0.9 is considered to indicate high diagnostic accuracy, AUC: 0.9-0.7 is considered relatively diagnostic accuracy, and AUC: 0.7-0.5 is considered low diagnostic accuracy^22^.

### Prognostic significance of LY6H across cancers

We conducted an investigation into the correlation between LY6H expression levels and prognosis, with a specific focus on survival association analysis of overall survival (OS), disease-specific survival (DSS), and progression-free interval (PFI) for each tumor type. Initially, employing COX proportional hazards model analysis, we observed significant associations between LY6H expression level and OS in KIPAN (P<0.001), LIHC (P<0.001), READ (P=0.005), THCA (P=0.01), SARC (P=0.03), KIRC (P=0.03), GBMLGG (P<0.001), LGG (P<0.001), PAAD (P=0.004) and ALL-R (P=0.005). Secondly, considering GBMLGG, LGG, PAAD, ALL-R, ALL, DLBC, THYM, PRAD, PCPG, LAML, TGCT, WT, ACC, LAML, NB, COAD as examples; LY6H was identified as a low-risk factor while it served as a high-risk factor for other cancer types. In addition to this finding, Kaplan-Meier survival analysis demonstrated that patients with LUAD, LIHC, KIRP, and CHOL exhibited shorter OS when expressing high levels of LY6H; conversely, in patients with GBM, having higher LY6H expression was associated with better OS.

The COX proportional risk model analysis revealed a significant correlation between LY6H expression and the prognosis of patients with LIHC, CESC, ACC, and PAAD in DFI. Additionally, Kaplan-Meier survival analysis demonstrated that high LY6H expression was associated with unfavorable DFI outcomes in patients with CESC, BRCA, LIHC, and KIRC. Conversely, in LGG patients, high LY6H expression was linked to improved DFI.

The expression of LY6H in DSS was significantly correlated with KIRAN, LIHC, THCA, BRCA, KIRP, COADREAD, SARC and KIRC. Kaplan-meier survival analysis revealed that high LY6H expression was associated with poor DSS outcomes in patients with LIHC, COAD, GBM, CHOL and OV. Conversely, in patients with LGG, high LY6H expression was associated with improved DSS.

### Relationship between LY6H expression and immune cell infiltration

**Figure 7:**
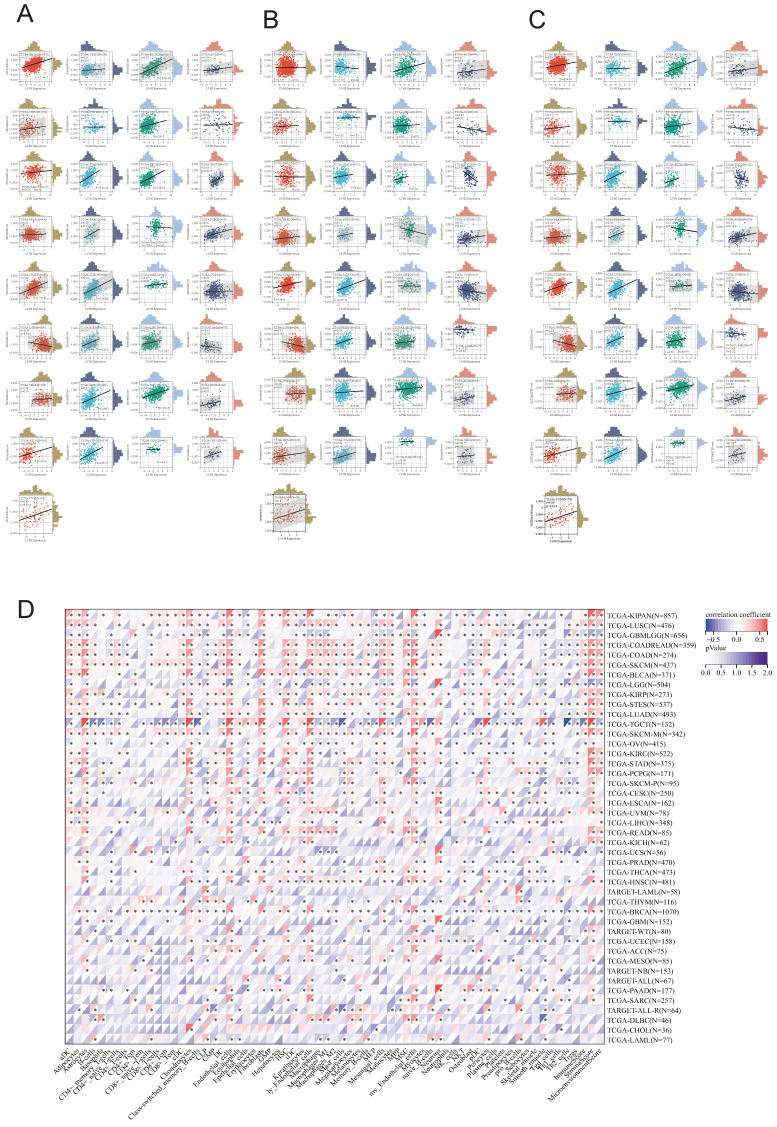
Relationship between LY6H expression and immune infiltration. **(A)** Scatter plot of correlation of stromalscore based on ESTIMATE algorithm for 33 cancers. **(B)** Scatter plot of correlation of immunescore for 33 cancers based on ESTIMATE algorithm. **(C)** Correlation scatter plot of estimatescore based on ESTIMATE algorithm for 33 cancers. **(D)** Heat map of correlation between LY6H expression and 67 immune cells in 33 cancers.

We utilized the ESTIMATE algorithm to compute the stromalscore, immunescore, and estimatescore of LY6H expression across 33 cancer types, subsequently generating a correlation scatter plot. The findings revealed that in terms of stromalscore (Figure 7A), LY6H expression exhibited positive correlations with BRCA, CESC, BLCA, ACC, UCEC, THCA, STAD, SKCM, TGCT, READ, PCPG, LUAD, LUSC, MESO, KIRP, LIHC, HNSC, KIRC, KICH, ESCA, COAD, CHOL, UVM; while displaying a negative correlation with LGG. Concerning immune score analysis (Figure 7B): LY6H expression demonstrated positive associations with BLCA, STAD, SKCM, PRAD, PCPG, LUAD, LUSC, KIRP, LIHC, ESCA, COAD, UVM; conversely exhibiting negative correlations with UCS TGCT PAAD OV and LGG. In terms of estimatescore evaluation (Figure 7C): LY6H expression was positively correlated with BRCA, BLCA, THCA, STAD, SKCM, PRAD, READ, PCPG, LUAD, LUSC, KIRP, LIHC, HNSC, KIRC, KICH, ESCA, COAD, UVM; whereas it displayed negative correlations with TGCT, OV and LGG.

**Figure 8:**
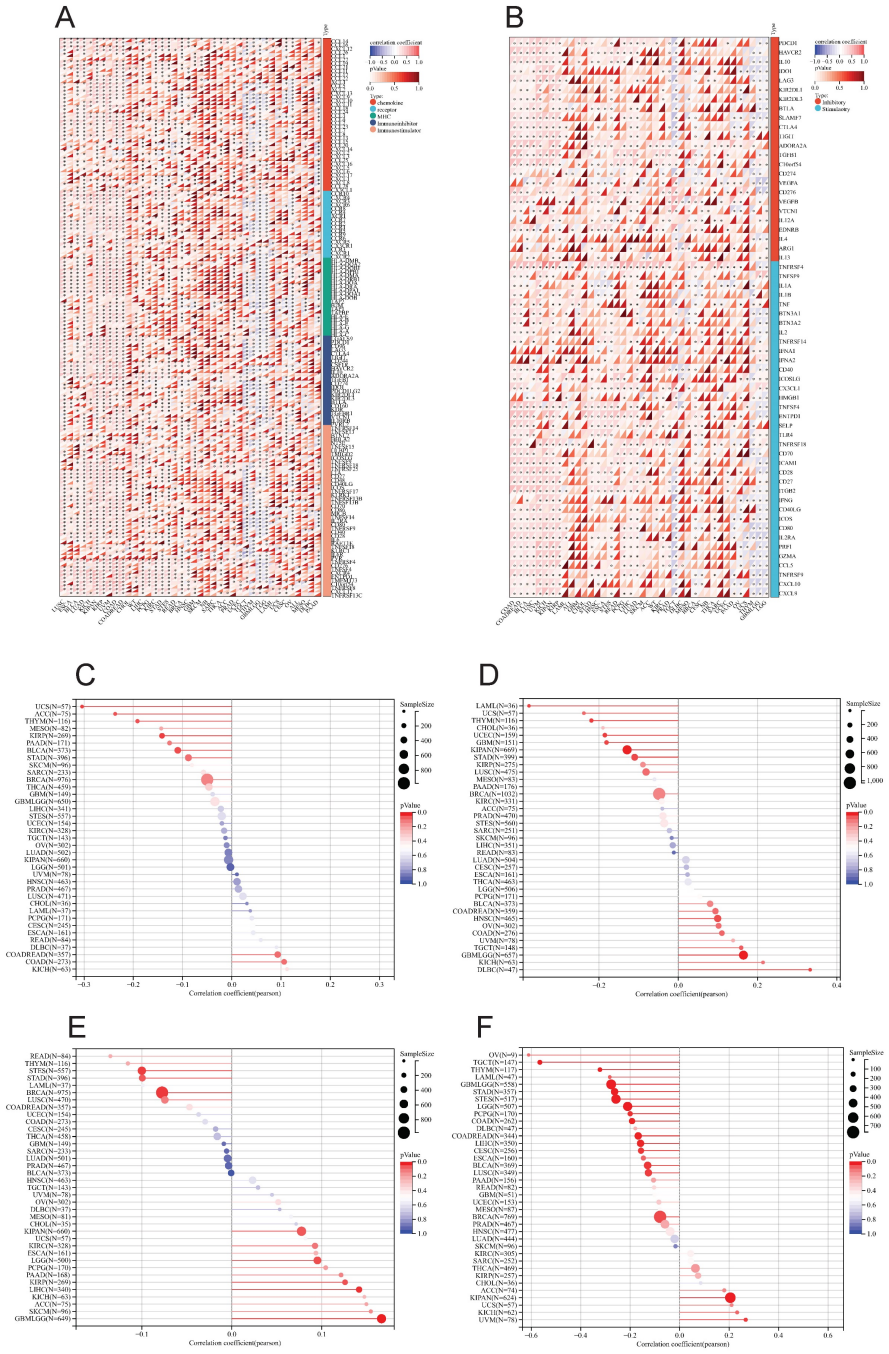
** (A)** Heat map of LY6H association with immunomodulatory genes in various cancers. **(B)** Heat map of LY6H association with immune checkpoint genes in various cancers. **(C)** Association of LY6H with TMB in various cancers. **(D)** association of LY6H with MSI in various cancers. **(E)** association of LY6H with MATH in various cancers. **(F)** association of LY6H with tumor stemness in various cancers.

The correlation heat maps (Figure 7D) we have generated demonstrate a significant association between LY6H expression and immune cell infiltration levels across the majority of cancer types. The correlation analysis of LY6H expression and immunomodulatory genes revealed a significant association between LY6H expression and immunomodulatory genes across various cancer types (Figure 8A), including pivotal immune genes such as IL2RA (also known as CD25), IL-2, and CTLA4^23^. The analysis of immune checkpoint genes revealed a significant correlation between LY6H expression and multiple key immune checkpoint genes across various cancer types (Figure 8B), including PD-1, PD-L1, CTLA-4, LAG-3, TIGIT, BTLA, TIM-3, AA2R, CEACAM1, SIRP-α, and CD200R.

### Association of LY6H with TMB, MSI, MATH, and tumor stemness

We calculated the correlation between LY6H expression and TMB in each tumor, and observed a significant negative correlation in 5 tumors, such as KIRP (P=0.02), THYM (P=0.03), UCS (P=0.02), BLCA (P=0.03), ACC (P=0.04). For MSI, we observed a significant association in 9 tumors, among which there was a significant positive association in 3 tumors, such as GBMLGG (P=0.001), HNSC (P=0.03), DLBC (P=0.02), and a significant negative association in 6 tumors. Such as: GBM (P=0.02), LAML (P=0.02), KIPAN (P=0.001), STAD (P=0.02), UCEC (P=0.01), THYM (P=0.01). For MATH, we observed a significant association in 8 tumors, including a significant positive association in 5 tumors, such as GBMLGG (P=0.001), LGG (P=0.03), KIRP (P=0.03), KIPAN (P=0.04), LIHC (P=0.008), and a significant negative association in 3 tumors. For example, BRCA (P=0.01), STES (P=0.01), STAD (P=0.04).

The correlation analysis between tumor stemness and LY6H expression revealed significant correlations in 16 tumors. Among them, two tumors (KIPAN: p < 0.001; UVM: P=0.017) showed a significant positive correlation, while the remaining 14 tumors exhibited a significant negative correlation. Notably, GBMLGG (p < 0.001), LGG (p < 0.001), CESC (P=0.012), COAD (P=0.001), COADREAD (P=0.001), BRCA (P=0.029), STES (p < 0.001), STAD (p < 0.001), LUSCP (P=0.018), THYM (p < 0.001), LIHC (P=0.003), TGCT (P=0.003), PCPG (p < 0.001), and BLCA (P=0.012) were among the significantly correlated tumors.

### Relationship between LY6H expression and methylation

**Figure 9:**
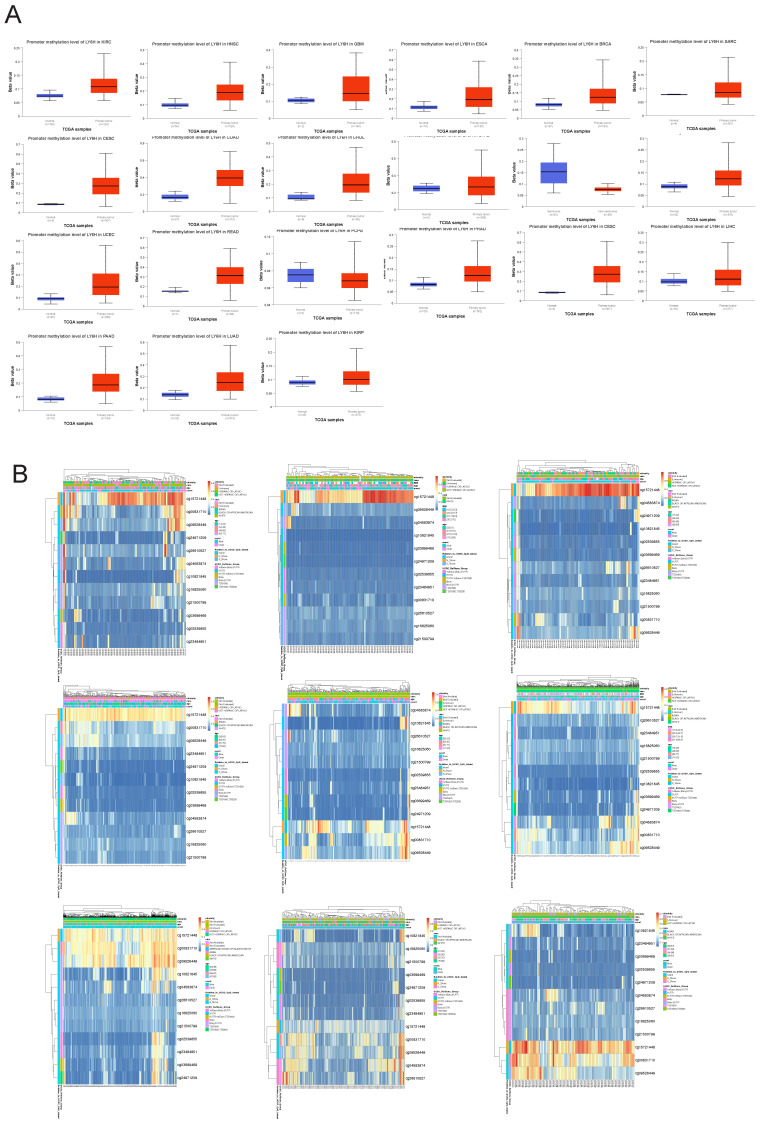
** (A)** Promoter methylation levels of LY6H in cancer. **(B)** Heat map of LY6H methylation sites in cancer.

To investigate the underlying mechanisms driving tumor progression, we utilized the UALCAN online tool to assess the methylation levels of LY6H promoter in distinct cohorts of cancer patients and healthy individuals. Hypermethylation was defined as beta values ranging from 0.7 to 0.5, while hypomethylation was characterized by beta values between 0.3 and 0.25. Figure 9A demonstrates a significant reduction in LY6H promoter methylation levels among the twenty tumor groups compared to the normal group. In addition, we utilized the MethSurv database to examine the correlation between DNA methylation and LY6H expression (Figure 9B). Our methylation heat map reveals a predominant decrease in methylation levels at most DNA methylation sites associated with LY6H across various cancers. Of particular interest, the cg15721488 methylation site linked to LY6H expression exhibits hypermethylation in nearly all cancer types, highlighting its significance as a noteworthy methylated site.

### Construction of PPI and GGI networks

**Figure 10:**
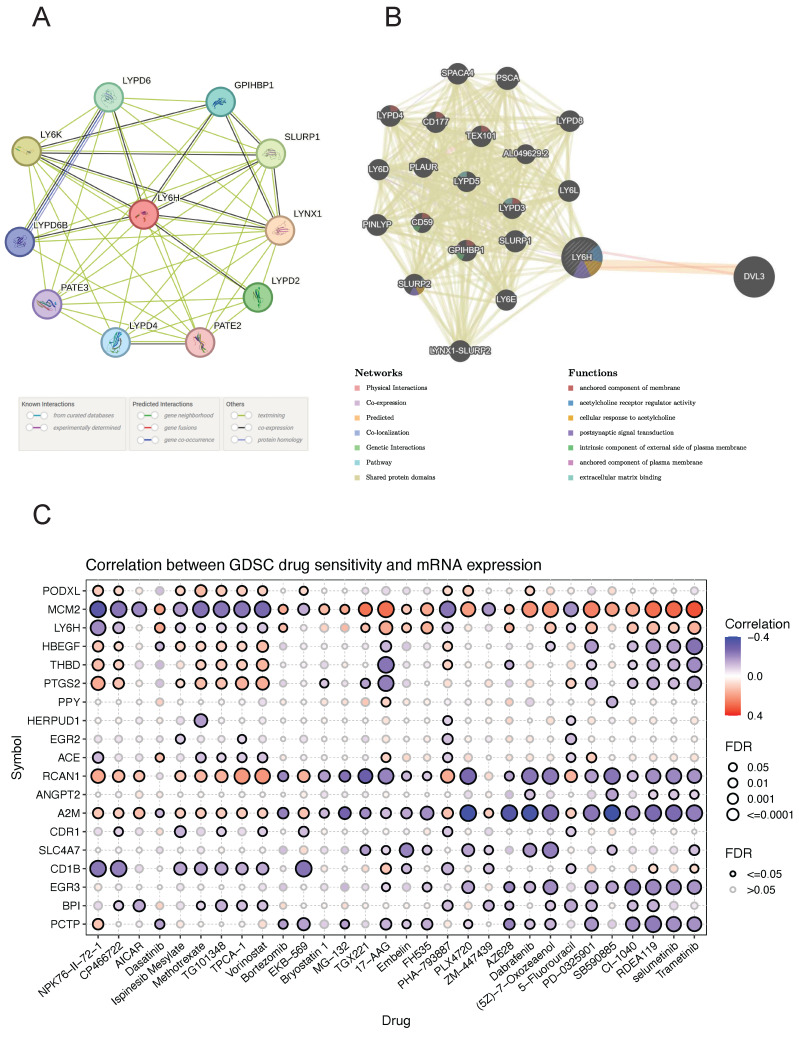
** (A)** GGI network associated with the LY6H gene. **(B)** PPI network associated with LY6H protein. **(C)** Correlation heatmap of LY6H and its associated genes in drug susceptibility.

The PPI networks of LY6H and 10 associated proteins (Figure 10A), namely LYPD6, GPIHBP1, SLURP1, LYNX1, LYPD2, PATE2, LYPD4, PATE3, LYPD6B and LYPD6B were acquired through the utilization of STRING database analysis. GeneMANIA was utilized to construct gene-gene interaction networks for LY6H and its homologous genes (Figure 10B). The findings revealed a close association between the top 20 genes with the highest mutation frequency and LY6H, with DVL3 exhibiting the most significant correlation. Furthermore, functional analysis demonstrated a significant relationship between LY6H and its homologous genes in terms of membrane anchoring components, regulatory activity of acetylcholine receptors, cellular response to acetylcholine, postsynaptic signal transduction, intrinsic components of the outer plasma membrane, plasma membrane anchoring components, and extracellular matrix binding.

### Drug sensitivity analysis

We identified drug resistance to LY6H and its associated genes, including PODXL, MCM2, HBEGF, THBD, PTGS2, and PPY through analysis of the GSCA database (Figure 10C). A positive correlation indicates that high expression of the LY6H gene confers resistance to the drug, while a negative correlation suggests that high expression of LY6H makes cells more sensitive to the drug. As shown in the correlation heat map, increased expression of LY6H enhances sensitivity to NPK76-Ⅱ-72-1, CP466722 and Ispinesib Mesylate; Methotrexate; TG101348; TPCA-1; vorinostat; PHA-793887; 5-Fluorouracil among others. Conversely, elevated expression of LY6H leads to drug resistance against Dasatinib; Bortezomib; TGX221; 17-AA; Embelin; FH535; AZ628; (5Z)-7-Oxozeaenol; PD-0325901; CI-1040; RDEA119, selumetinib Trametinib and other drugs.

### Molecular subtypes and immune subtypes

**Figure 11:**
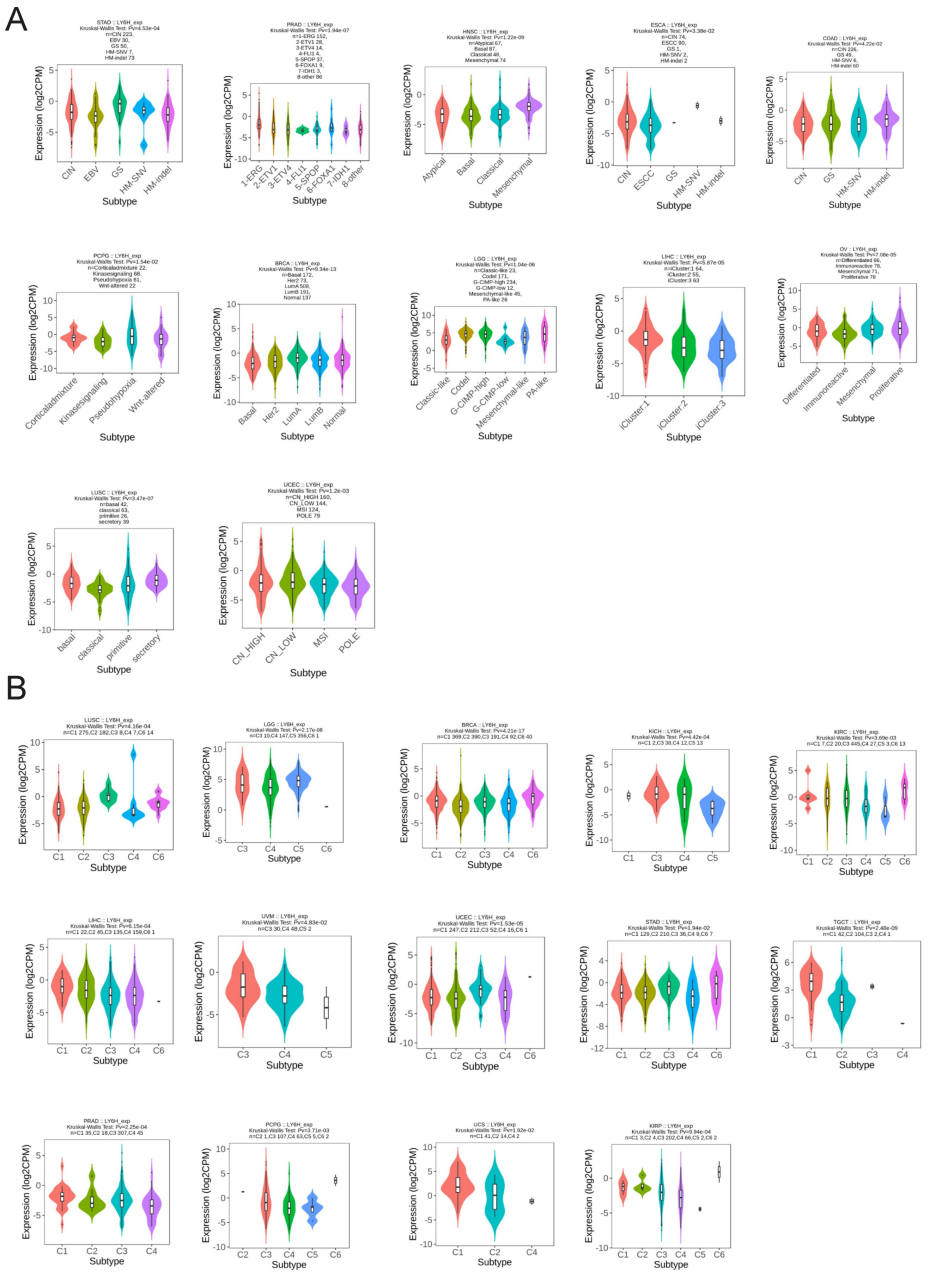
Molecular and immunophenotypes of LY6H in pan-cancer in the TISIDB database.** (A)** 12 molecular subtypes of LY6H in cancer; **(B)** 14 immunophenotypes of LY6H in cancer.

We utilized the TISIDB database to conduct an analysis on the expression patterns of molecular and immune subtypes of LY6H across various cancer types (Figure 11A). Our findings revealed that LY6H exhibited expression in twelve distinct molecular subtypes of cancer. Notably, LY6H displayed the highest expression levels in the GS subtype of STAD, 1-ERG subtype of PRAD, Mesenchymal subtype of HNSC, and CIN subtype of ESCA. Additionally, COAD demonstrated elevated expression levels for HM-SNV and HM-indel, PCPG showed increased Pseudohypoxia expression, BRCA exhibited LumA overexpression, while LGG presented with heightened Codel and G-CIMP-high expressions. Moreover, LIHC showcased maximum iGluster:1 subtype expression; OV displayed higher Differentiated and Proliferative subtype expressions; whereas LUSC demonstrated elevated bacal and secretory subtype expressions. Lastly, UCEC indicated higher expression levels within CN-HIGH and CN-LOW subtypes. The immune subtypes encompass C1: wound healing, C2: interferon-gamma dominant, C3: inflammatory, C4: lymphocyte depletion, C5: immune quiet, and C6: transforming growth factor-β dominant. It is evident that the expression of LY6H exhibits correlation with 14 cancer types including LUSC, LGG, BRCA, KICH, KIRC, LIHC, UVM, UCEC, STAD, TGCT, PRAD, PCPG, UCS and KIRP (Figure 11B).

### Genetic alteration and single cell function analysis

**Figure 12:**
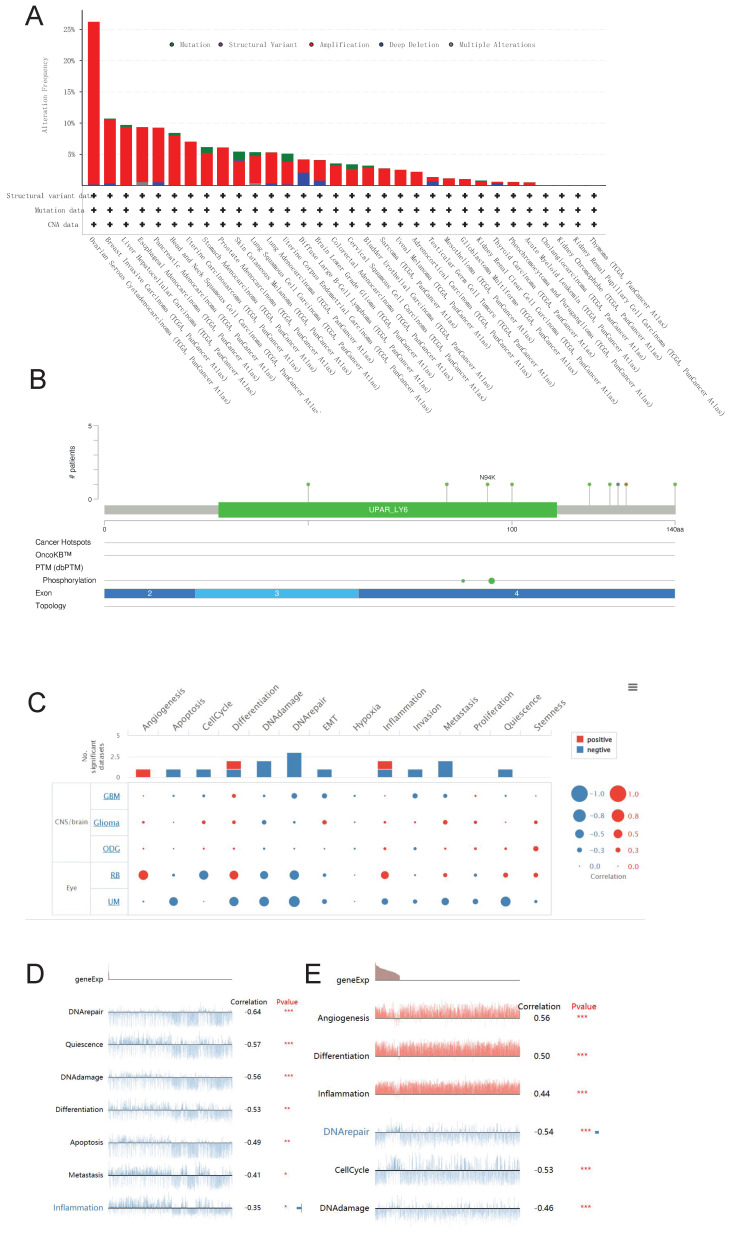
Genetic alterations and single-cell functional analysis of LY6H. **(A)** the frequency of change in LY6H. **(B)** Visual summary of changes in LY6H queries from cBioPortal by OncoPrint. **(C-E)** LY6H functions at the single-cell level.

We utilized the cBioPortal database to conduct an analysis on the genetic alterations of LY6H across various cancer types. As visually depicted in Figure 12A, a predominant majority of cancer types exhibited amplifications as the primary form of genetic alteration. Notably, Diffuse Large B-Cell Lymphoma displayed a prevalence of both mutations and amplifications in gene changes. Furthermore, Ovarian Serous Cystadenocarcinoma demonstrated the highest frequency (exceeding 25%) of gene alterations. Our analysis also revealed that somatic mutations in LY6H were predominantly deletions (Figure 12B).

We investigated the role of LY6H at the single-cell level using CancerSEA(Figure 12C). Our findings demonstrate a significant positive correlation between LY6H gene expression and angiogenesis, while revealing a significant negative correlation with apoptosis, cell cycle regulation, DNA damage response and repair mechanisms, epithelial-mesenchymal transition (EMT), invasion, metastasis and quiescence. In uveal melanoma (UM), we observed a negative association between LY6H expression levels and DNA repair capacity, cellular quiescence status, DNA damage response pathways as well as differentiation potential, apoptosis susceptibility and inflammatory signaling cascades. Conversely in retinoblastoma (RB), we found that LY6H was positively correlated with angiogenic processes along with differentiation programs and inflammation-related pathways; whereas it exhibited an inverse relationship with DNA repair efficiency as well as cell cycle progression.

### Interacting chemicals and genes of LY6H

The CTD database recorded a total of 55 chemicals associated with LY6H, out of which 18 were identified as potential up-regulators and 16 as potential down-regulators of LY6H expression. Furthermore, the impact of 11 chemicals on LY6H expression was confirmed, although the specific effects remain unclear (Table 1). Additionally, through chemical association analysis, we identified the top 20 gene relationships with LY6H. Notably, strong correlations were observed between LY6H and FAM131C, NIPA2, GRM7, DMBX1, CHADL, KCNH4, and CDHR2 (Table 2).

### Identification of DEGs and Functional enrichment analysis in LIHC

**Figure 13:**
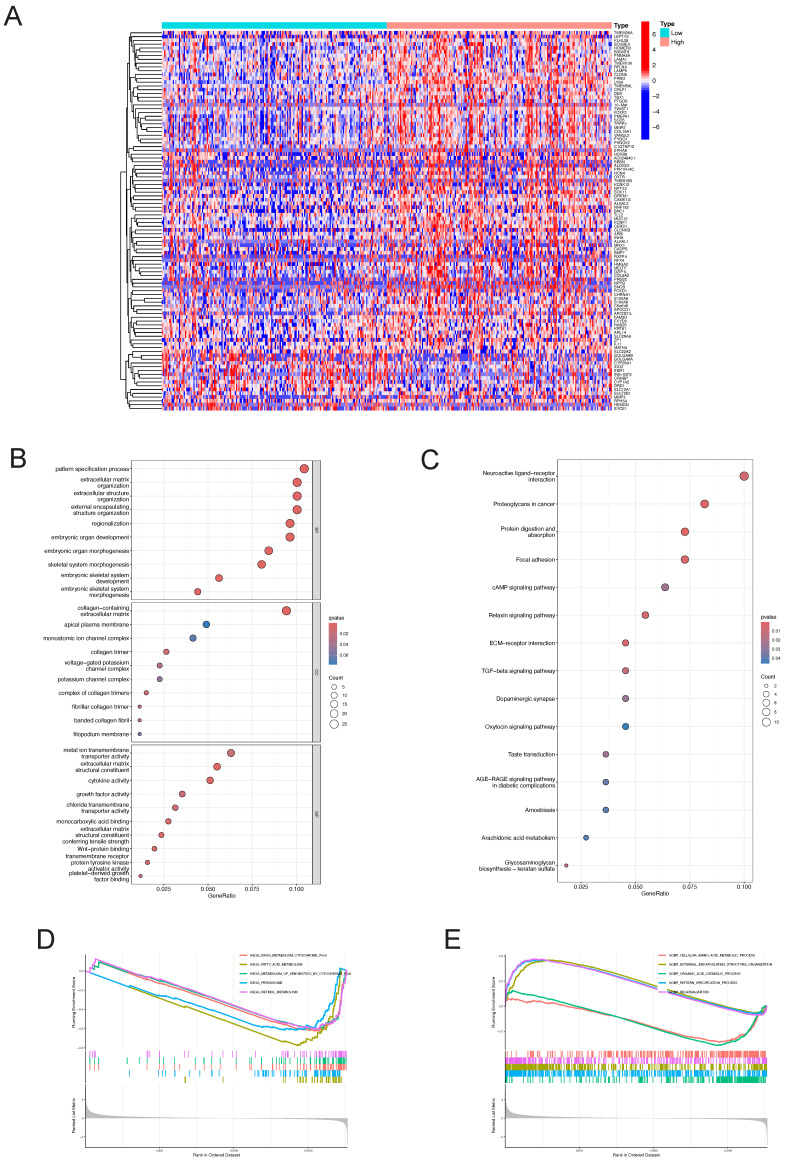
Differential gene identification of LY6H and enrichment analysis in LIHC **(A)** heat map of co-expressed genes in the LY6H high/low expression group. **(B)** GO enrichment analysis. **(C)** KEGG classification analysis. **(D, E)** GSEA enrichment analysis.

The "pheatmap" R package was utilized to generate a heatmap illustrating the differential gene expression in the LY6H high/low expression group (Figure 13A). This visualization allowed for a comprehensive assessment of the expression levels of each differentially expressed gene within LY6H. We utilized GO terminology and the KEGG pathway database for gene annotation. Through analysis using the GO database (Figure 13B), we obtained several significant results related to embryonic organ development, embryonic organ morphogenesis, embryonic skeletal system development, skeletal ine activity, growth factor activity, Wnt-protein binding transmembrane receptor, protein tyrosine kinase activator activity, and platelet-derived growth factor binding. We have conducted gene annotation using the KEGG database, and have identified several noteworthy functions and pathways (Figure 13C), including Neuroactive ligand-receptor interaction, Proteoglycans in cancer, Focal adhesion, cAMP signaling pathway, Relaxin signaling pathway, ECM-receptor interaction, TGF-beta signaling pathway and Oxytocin signaling pathway.

Additionally, employing GSEA pathway analysis revealed several pivotal signaling pathways in the LY6H low expression group (Figure 13D), including drug metabolism cytochrome P450, fatty acid metabolism, metabolism of xenobiotics by cytochrome P450, peroxisome, and retinol metabolism. Through GSEA functional analysis, we identified several key functions enriched in the LY6H high expression group (Figure 13E), including regionalization, pattern specification processes, and organization of external encapsulating structures. In contrast, the LY6H low expression group showed enrichment for cellular amino acid metabolic processes and organic acid catabolic processes.

## Discussion

Cancer has emerged as a critical determinant of human survival and quality of life, making it the focal point of current medical research. Consequently, the identification of an appropriate cancer treatment modality assumes paramount importance in extending human lifespan. Leveraging patient information from TCGA, GTEx, UALCAN, cBioportal, UCSC, CTD, cancerSEA databases, we conducted an extensive analysis on the differential expression of LY6H across 33 human cancers to explore its associations with diagnosis, prognosis, immune infiltration, methylation patterns, genetic alterations, tumor stemness characteristics and chemical substances. Our findings aim to identify potential broad-spectrum biomarkers for cancer diagnosis by highlighting the significant up-regulation of LY6H in various cancer types and its potential utility in early detection.

Previous studies have shown that LY6H, like other LY6 family genes, is significantly expressed in a variety of cancers, including prostate cancer, bladder cancer, ovarian cancer, and skin cancer^11^, ^19^, ^24^, ^25^. The recent functional investigations of the LY6H gene on human chromosome 8 have revealed that LY6H and its family genes are implicated in cancer progression and immune infiltration, serving as significant biological indicators for unfavorable cancer prognosis^5^, ^21^. The stem cell antigen-1(Sca-1) represents the inaugural member of the Ly6 gene family in mice. Sca-1 has been identified as a distinctive marker for resident tissue stem cells and is also acknowledged as a population of cancer-initiating cells in murine models of various cancer types, including breast, prostate, and lung cancer^26^-^28^.

We conducted a comprehensive analysis and screening of numerous genes, leading to the identification of LY6H with its unique detection performance. By excluding insufficient normal samples, duplicate samples, and samples with 0 mRNA expression, we observed significant differences in the expression levels of LY6H between tumor and normal tissues across 16 types of cancer. Specifically, elevated expression of LY6H was detected in BLCA, CHOL, GBM, HNSC, KICH, KIRC, KIRP, LIHC, LUAD, PCPG. Conversely, LY6H exhibited lower expression levels in CESC, COAD, ESCA, READ, STAD and UCEC compared to their respective normal tissues. Moreover, in the matched tumor samples, LY6H expression was significantly elevated in BLCA, CHOL, HNSC, KICH, KIRC, KIRP, LIHC, CESC and THCA in nine cancers compared with the corresponding normal samples. In contrast, in paired tumor samples, LY6H expression was significantly reduced in STAD. The collective findings provide further evidence of the up-regulation of LY6H expression across various cancer types, thereby indicating the promising potential of LY6H in cancer diagnosis.

The previous studies have demonstrated the paramount clinical implications of early cancer detection, enabling timely treatment options for cancer patients. However, currently there is a lack of suitable biological indicators to serve as references for early cancer diagnosis^29^. Therefore, we conducted an investigation into the variations in LY6H expression across diverse clinical parameters, encompassing pathological stage, TNM stage, clinical stage, histological grade, WHO grade, gene status, vascular invasion, AFP level and other relevant factors. These clinical parameters hold significant diagnostic value in specific cancer types. Based on our findings, LY6H expression was significantly upregulated in the early stages of six tumors, while it exhibited a significant reduction in the early stages of seven tumors. Regarding histological grade, LY6H expression showed a significant increase at the initial histological stage in four tumors and decreased significantly at the early histological stage in three tumors. Previous studies have demonstrated that cancers diagnosed at an earlier stage generally have a more favorable prognosis than those diagnosed at advanced stages^30^-^32^. The expression of LY6H is higher in certain cancers at the early stage, such as CHOL (histological grade G2, Pathological stage I&II), BRCA (Pathological stage I), LIHC (Pathological stage I, histological grade G1), KIRP (Pathological stage I, histological grade G1), KICH (Pathological stage I, histological grade G1). HNSC (Pathological stage II, histological grade G2). This finding can provide valuable guidance for clinicians in disease diagnosis. Previous studies have demonstrated a correlation between IDH status in LGG and Tumor infiltrating lymphocytes (TILs) as well as programmed death ligand 1(PD-L1). IDH-wt gliomas exhibit more pronounced TIL infiltration and higher PD-LI expression compared to IDH-mut cases^33^. These findings suggest that the immunoregulatory treatment strategies for glioma patients should be tailored based on their IDH status. In our study, we observed elevated levels of LY6H expression in IDH-mut compared to IDH-wt cases. Therefore, our study can serve as a valuable reference for clinicians involved in immunotherapy. With regard to BRCA, there is a positive correlation between increased LY6H expression and the activity of ER, PR, and HER2 genes. Compelling literature suggests that assessing the expression status of these genes in residual tumors after neoadjuvant therapy could enhance personalized adjuvant treatment strategies. Furthermore, previous studies have demonstrated associations between the activity of ER, PR, and HER2 genes with axillary lymph node metastasis as well as brain metastasis^34^-^36^. In addition, in LIHC, elevated LY6H exhibited a robust correlation with vascular infiltration and AFP levels. The measurement of AFP level demonstrates excellent diagnostic performance for early-stage HCC and post-treatment progression, making it the most commonly utilized biological marker in clinical settings^37^-^39^. The AUC of the ROC curve further validated the exceptional diagnostic performance of LY6H across multiple cancer types.

The association between LY6H expression level and prognosis was investigated by performing survival association analysis for each type of cancer, including overall survival (OS), disease-free interval (DFI), and disease-specific survival (DSS), using Kaplan-Meier survival curves. Based on these findings, high LY6H expression was found to be significantly associated with a poorer prognosis in patients with LUAD, LIHC, KIRP, CHOL, CESC, BRCA, COAD, GBM and OV.

By investigating the association between LY6H gene expression and stromalscore, immunescore, and estimatescore, we observed a significant positive correlation between LY6H expression and these three scores in the majority of cancer types. Furthermore, LY6H exhibits interactions with both tumor cells and immune cells. The utilization of Immunoscore as an indicator can effectively assess the prognosis, recurrence risk, metastasis potential, and drug resistance in cancer patients. Meanwhile, through investigating the correlation between LY6H and immune cell infiltration, we observed a significant association of LY6H with 67 distinct immune cell types in various cancers, encompassing B cells, CD4^+^T cells, CD8^+^T cells, neutrophils, macrophages, and dendritic cells that play pivotal roles within the tumor microenvironment. Previous research has demonstrated that the composition, localization, and abundance of immune cells in the tumor microenvironment are closely linked to cancer progression^40^. Gaining insights into the specific subpopulations of quiescent immune cells present in the tumor microenvironment can serve as a valuable reference for advancing cancer immunotherapy^41^. We also observed a significant correlation between LY6H expression and immunomodulatory genes, including key genes such as IL2RA, IL-2, and CTLA438. Additionally, immune checkpoint genes such as PD-1, PD-L1, CTLA-4, LAG-3, TIGIT, BTLA, TIM-3, AA2R, CEACAM1,SIRP-α,and CD200R were found to be significantly associated. Previous studies have demonstrated that polymorphisms in the immunomodulatory gene IL2RA are linked to an increased risk of lung cancer and the development and progression of acute myeloid leukemia in the Chinese Han population^42^. Furthermore, numerous reports have emphasized the significance of the immunomodulatory gene IL-2 in cancer treatment and autoimmune diseases^43^-^45^. The investigation of immune checkpoint genes serves as a crucial point of reference for the advancement of immune checkpoint inhibitors and represents a groundbreaking development in cancer therapy^46^. Actively exploring novel immune checkpoint genes is imperative for effective cancer treatment. For instance, the research conducted by Long Long et al. demonstrated that combination immunotherapy involving anti-LAG-3 and anti-PD-1 exhibited remarkable efficacy against PD-1 resistance, underscoring the pivotal role played by immune checkpoint genes in tumor management^47^. Exploring the relationship between LY6H and drug sensitivity and drug resistance can provide new ideas for the treatment of tumors. Different immune subtypes and molecular subtypes can also be used as an entry point for new ideas for tumor treatment. Our study can provide valuable insights into cancer immunotherapy.

By examining the correlation between LY6H expression and tumor mutational burden(TMB), microsatellite instability (MSI), and mutation-associated thermodynamic stability(MATH) genes, we observed a significant association between LY6H expression and TMB across multiple cancer types. Considering previous evidence suggesting that high TMB is linked to improved survival following immune checkpoint inhibitor (ICI) therapy in various cancers, it is noteworthy that higher TMB has been associated with poorer prognosis in several malignancies^48^. Moreover, elevated TMB in ICI treated patients has been correlated with prolonged survival. However, limited research exists on the relationship between TMB and the immune microenvironment. Additionally, relevant studies have demonstrated that MSI status can predict resistance to cancer drugs, making it an important predictor of immunotherapy-based treatment efficacy^49^. Through the analysis of LY6H expression and tumor stemness, we have identified significant correlations between 16 types of cancer and tumor stemness. Previous studies have demonstrated the crucial role of tumor stemness in cancer initiation and progression, as well as a general inverse relationship between cancer stemness and anti-cancer immunity. Consequently, our findings provide insights into the involvement of LY6H in the immune microenvironment, serving as a valuable reference for immunotherapy^50^.

Our study revealed a significant correlation between LY6H expression and DNA hypomethylation, indicating poor prognosis. Previous studies have identified global hypomethylation and specific promoter hypermethylation as canonical epigenetic changes associated with genomic instability and tumor suppressor gene silencing^51^. In our analysis of 20 cancer types, we found that hypomethylation of the LY6H promoter was associated with higher LY6H expression, suggesting a potential role in tumor progression^52^. The causes of abnormal hypermethylation at cg15721488 can be further explored in the future. Additionally, we have identified compounds that potentially modulate the expression of LY6H and constructed a gene interaction network comprising the 20 genes most closely associated with LY6H through chemical binding.

From the results interpreted by the cBioPortal platform, we know that LY6H is mutated in most forms of tumors. Among them, Diffuse Large B-Cell Lymphoma and Ovarian Serous Cystadenocarcinoma have the highest incidence, suggesting that attention should be paid to the relationship between LY6H gene mutation and blood system and female reproductive system. In addition, our single-cell functional analysis revealed a significant positive correlation between LY6H gene expression and angiogenesis, as well as a negative correlation with apoptosis, cell cycle regulation, DNA damage response and repair mechanisms, epithelial-mesenchymal transformation (EMT), invasion, metastasis, and quiescence.

Through KEGG enrichment analysis, we identified several significant pathways, including Neuroactive ligand-receptor interaction, Proteoglycans in cancer, Focal adhesion, cAMP signaling pathway, Relaxin signaling pathway, ECM-receptor interaction, TGF-β signaling pathway, and Oxytocin signaling pathway. Numerous studies have shown that these pathways are closely related to the occurrence and development of cancer^53^-^55^.

In our study, we investigated the role of LY6H in TCGA human pan-cancer, encompassing diagnostic, clinical, and immunological features. Specifically, we discovered that LY6H served as an unfavorable prognostic indicator. LY6H exhibited a strong correlation with immunotherapy-related characteristics such as immune cells, immunomodulatory genes, immune checkpoints, tumor stemness, and TMB, thereby highlighting its potential as an immunotherapy predictor. However, it is important to note that our study only validated the differential expression of LY6H in HCC, necessitating further investigation into the specific role of LY6H in each tumor type. Additionally, this paper delved into methylation levels of LY6H along with drug sensitivity analysis, mutation analysis and chemical analysis to provide insights into elucidating the mechanism underlying tumor development.

## Supplementary Material

Supplementary table.

## Figures and Tables

**Figure 4:**
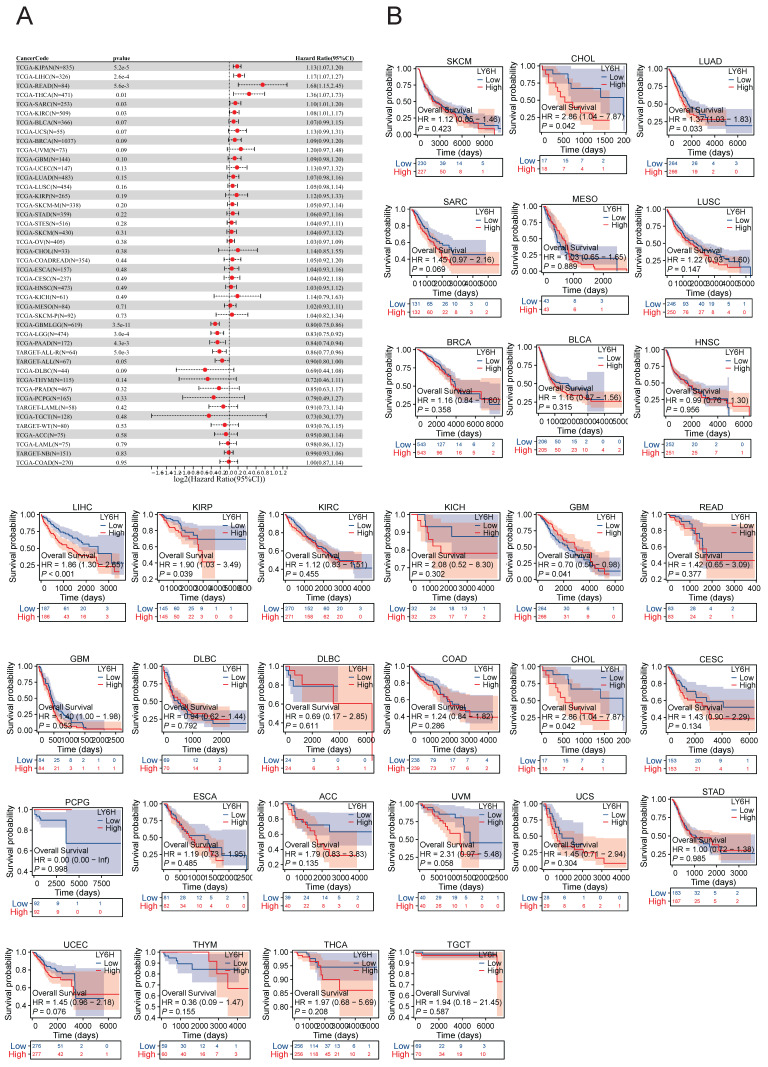
Association between LY6H expression and overall survival (OS). **(A)** Forest plot of OS associations in cancers in 33. **(B)** Kaplan-Meier analysis of the association between LY6H expression and OS.

**Figure 5:**
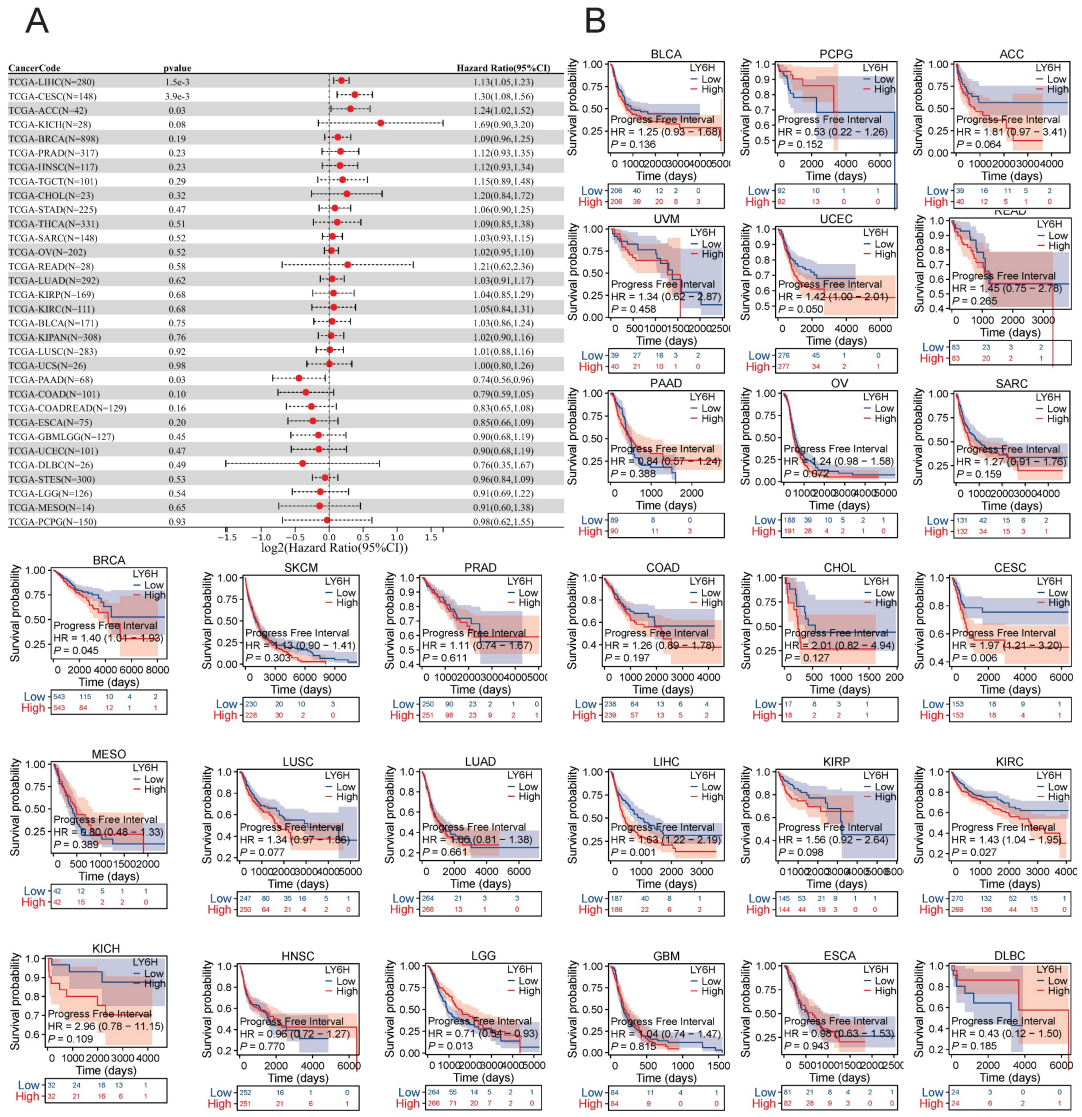
Association between LY6H expression and progression-free interval (PFI).** (A)** Forest plot of PFI associations in cancers in 33. **(B)** Kaplan-Meier analysis of the association between LY6H expression and PFI.

**Figure 6:**
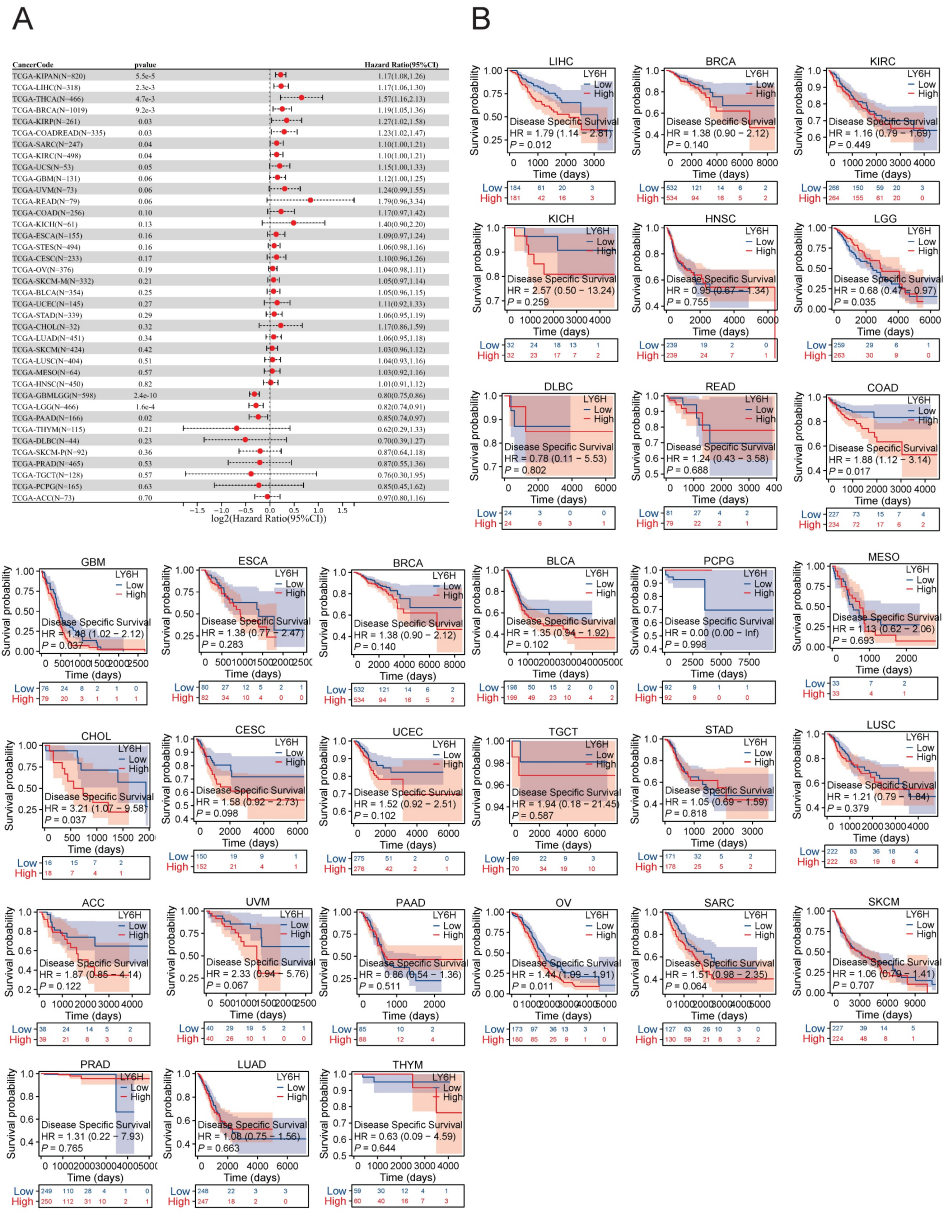
Association between LY6H expression and disease-specific survival (DSS). **(A)** Forest plot of DSS associations in cancers in 33. **(B)** Kaplan-Meier analysis of the association between LY6H expression and DSS.

**Table 1:** Interacting chemicals of Ly6H from CTD

Chemical Name	ID	Interaction Actions	Chemical Name	ID	Interaction Actions
2,3,7,8-tetrachlorodibenzofuran	C014211	increases^expression	Dioxins	D004147	affects^methylation
Acetaminophen	D000082	affects^expression	Dronabinol	D013759	decreases^expression
Acrylamide	D020106	decreases^expression	Fenretinide	D017313	decreases^expression
Aflatoxin B1	D016604	decreases^methylation	furan	C039281	decreases^methylation
Amphetamine	D000661	increases^expression	furan	C039281	increases^expression
Asbestos, Crocidolite	D017638	increases^expression	Lead	D007854	affects^expression
AZM551248	C547126	decreases^expression	lipopolysaccharide, E coli O55-B5	C482199	decreases^expression
Benzo(a)pyrene	D001564	decreases^expression	Methamphetamine	D008694	decreases^expression
Benzo(a)pyrene	D001564	decreases^methylation	Methoxychlor	D008731	affects^methylation
Benzo(a)pyrene	D001564	increases^methylation	mono-(2-ethylhexyl)phthalate	C016599	increases^expression
Benzo(a)pyrene	D001564	increases^methylation	N-Methyl-3,4-methylenedioxyamphetamine	D018817	increases^expression
bisphenol A	C006780	affects^expression	Plant Extracts	D010936	affects^cotreatment|decreases^expression
bisphenol A	C006780	affects^cotreatment|decreases^expression	Propylthiouracil	D011441	increases^expression
bisphenol A	C006780	increases^expression	Resveratrol	D000077185	affects^cotreatment|decreases^expression
bisphenol F	C000611646	affects^cotreatment|decreases^expression	Rotenone	D012402	decreases^expression
bisphenol F	C000611646	decreases^expression	sodium arsenite	C017947	increases^expression
bisphenol S	C543008	affects^cotreatment|decreases^expression	Tetrachlorodibenzodioxin	D013749	affects^expression
bisphenol S	C543008	decreases^expression	Tobacco Smoke Pollution	D014028	decreases^expression
Chlorpyrifos	D004390	increases^expression	Trichloroethylene	D014241	increases^expression
Cocaine	D003042	increases^expression	Triclosan	D014260	increases^expression
Cuprizone	D003471	decreases^expression	Valproic Acid	D014635	increases^expression
Cytarabine	D003561	decreases^expression	Valproic Acid	D014635	increases^methylation
decamethrin	C017180	increases^expression	-	-	-

**Table 2:** Relationship of LY6H with genes via chemical interaction, based on the CTD database

Gene	Similarity Index	Common Interacting Chemicals
FAM131C	0.32075472	17
NIPA2	0.31147541	19
GRM7	0.30882353	21
DMBX1	0.30769231	16
CHADL	0.30188679	16
KCNH4	0.30188679	16
CDHR2	0.30000000	18
C2CD4C	0.29824561	17
CCDC78	0.2962963	16
TLCD3B	0.29411765	15
EMX1	0.29310345	17
COL26A1	0.29230769	19
NPB	0.29032258	18
CCDC27	0.28947368	11
CCDC73	0.28888889	13
CABP7	0.28846154	15
TRIM72	0.28813559	17
HYDIN	0.28767123	21
PRR32	0.28571429	16
PTPRT	0.28571429	20

## Abbreviations

ACC: adrenocortical carcinoma
BLCA: bladder urothelial carcinoma
BRCA: breast invasive carcinoma
CESC: cervical squamous cell carcinoma and endocervical adenocarcinoma
CHOL: cholangiocarcinoma
COAD: colon adenocarcinoma
DLBC: lymphoid neoplasm diffuse Large B-cell lymphoma
ESCA: esophageal carcinoma
GBM: glioblastoma multiforme
HNSC: head and neck squamous cell carcinoma
KICH: kidney chromophobe
KIRC: kidney renal clear cell carcinoma
KIRP: kidney renal papillary cell carcinoma
LAML: acute myeloid leukemia
LGG: brain lower grade glioma
LIHC: liver hepatocellular carcinoma
LUAD: lung adenocarcinoma
LUSC: lung squamous cell carcinoma
MESO: mesothelioma
OV: ovarian serous cystadenocarcinoma
PAAD: pancreatic adenocarcinoma
PCPG: pheochromocytoma and paraganglioma
PRAD: prostate adenocarcinoma
READ: rectum adenocarcinoma
SARC: sarcoma
SKCM: skin cutaneous melanoma
STAD: stomach adenocarcinoma
TGCT: testicular germ cell tumors
THCA: thyroid carcinoma
THYM: thymoma
UCEC: uterine corpus endometrial carcinoma
UCS: uterine carcinosarcoma
UVM: uveal melanoma
