# Supplementary material for: Pan-cancer analysis suggests that LY6H is a potential biomarker of diagnosis, immunoinfiltration, and prognosis
Source: J Cancer. 2024 Aug 26;15(17):5515–39. doi: 10.7150/jca.98449 (PMC11414603; doi:10.7150/jca.98449)
Supplement: Supplementary file 1 — Supplementary table. [file jcav15p5515s1.pdf]

Table S1

The clinical parameters of 24 patients

Patients (ID)	Gender	Age	Tumor type	CNLC	BCLC stage	Child-pugh	AFP (ng/ml)	Vascular tumor thrombus	MVI	Smoking history	Alcohol history	HBV	hypertension	diabetes	Heart disease	History of surgery	Other Diseases
1	Male	48	Primary HCC	Ia	A	A	5.56	0	M0	1	1	1	0	0	0	0	0
2	Male	67	Primary HCC	IIIa	C	A	6.34	1	M0	1	1	1	0	0	0	0	0
3	Male	55	Primary HCC	Ib	A	A	11.26	0	M2	0	1	1	0	0	0	0	0
4	Male	43	Primary HCC	IIIa	C	A	5068	0	M0	1	1	1	0	0	0	0	0
5	Male	55	Primary HCC	IIb	B	A	40.90	0	M1	1	0	0	0	0	0	0	0
6	Male	53	Primary HCC	Ia	A	A	0	0	M0	1	1	1	0	0	0	0	0
7	Male	73	Primary HCC	IIa	B	A	64.60	0	M0	0	0	1	0	1	0	0	0
8	Male	36	Primary HCC	Ia	A	A	286.65	0	M0	1	1	1	0	0	0	0	0
9	Male	58	Primary HCC	Ib	B	A	329.38	0	M0	1	1	0	0	0	0	0	0
10	Male	60	Primary HCC	Ib	B	A	76102.96	0	M2	1	0	0	0	0	0	0	0
11	Male	53	Primary HCC	IIa	B	A	154.13	0	M1	0	0	0	1	0	0	0	0
12	Male	66	Primary HCC	Ib	A	A	56.97	0	M0	1	1	1	0	0	0	0	0
13	Male	63	Primary HCC	IIb	B	A	3568.69	0	M0	1	0	0	0	0	0	0	0
14	Female	66	Primary	Ia	A	A	2.05	0	M1	0	0	0	0	0	0	0	0

			HCC														
15	Male	57	Primary HCC	Ia	0	A	3.50	0	M0	1	1	1	0	0	0	0	0
16	Male	39	Primary HCC	Ia	0	A	319.32	0	M1	1	0	1	0	0	0	0	0
17	Male	35	Primary HCC	Ib	B	A	6963.40	0	M0	1	1	1	0	0	0	0	0
18	Male	33	Primary HCC	IIIa	C	A	295.50	0	M0	1	1	1	0	0	0	0	0
19	Male	52	Primary HCC	Ib	A	A	39.85	0	M0	1	1	1	1	0	0	0	0
20	Male	41	Primary HCC	Ib	B	A	160.76	0	M0	1	1	1	0	0	0	0	0
21	Female	61	Primary HCC	Ia	A	A	251.76	0	M0	0	0	1	0	0	0	0	0
22	Male	50	Primary HCC	Ib	C	A	86.09	0	M1	1	1	1	0	0	0	0	0
23	Male	58	Primary HCC	Ia	B	A	1.17	0	M0	1	0	1	0	0	0	0	0
24	Male	42	Primary HCC	IIa	B	A	2436.00	0	M0	1	1	0	0	0	0	0	0
